# HIV Proteins and Endothelial Dysfunction: Implications in Cardiovascular Disease

**DOI:** 10.3389/fcvm.2018.00185

**Published:** 2018-12-19

**Authors:** Appakkudal R. Anand, Gladys Rachel, Durgadevi Parthasarathy

**Affiliations:** ^1^L&T Microbiology Research Centre, Vision Research Foundation, Sankara Nethralaya, Chennai, India; ^2^Department of HIV/AIDS, National Institute for Research in Tuberculosis, Chennai, India

**Keywords:** HIV proteins, endothelial dysfunction, cardiovascular disease, gp120, Nef, Tat, atherosclerosis

## Abstract

With the success of antiretroviral therapy (ART), a dramatic decrease in viral burden and opportunistic infections and an increase in life expectancy has been observed in human immunodeficiency virus (HIV) infected individuals. However, it is now clear that HIV- infected individuals have enhanced susceptibility to non-AIDS (Acquired immunodeficiency syndrome)-related complications such as cardiovascular disease (CVD). CVDs such as atherosclerosis have become a significant cause of morbidity and mortality in individuals with HIV infection. Though studies indicate that ART itself may increase the risk to develop CVD, recent studies suggest a more important role for HIV infection in contributing to CVD independently of the traditional risk factors. Endothelial dysfunction triggered by HIV infection has been identified as a critical link between infection, inflammation/immune activation, and atherosclerosis. Considering the inability of HIV to actively replicate in endothelial cells, endothelial dysfunction depends on both HIV-encoded proteins as well as inflammatory mediators released in the microenvironment by HIV-infected cells. Indeed, the HIV proteins, gp120 (envelope glycoprotein) and Tat (transactivator of transcription), are actively secreted into the endothelial cell micro-environment during HIV infection, while Nef can be actively transferred onto endothelial cells during HIV infection. These proteins can have significant direct effects on the endothelium. These include a range of responses that contribute to endothelial dysfunction, including enhanced adhesiveness, permeability, cell proliferation, apoptosis, oxidative stress as well as activation of cytokine secretion. This review summarizes the current understanding of the interactions of HIV, specifically its proteins with endothelial cells and its implications in cardiovascular disease. We analyze recent *in vitro* and *in vivo* studies examining endothelial dysfunction in response to HIV proteins. Furthermore, we discuss the multiple mechanisms by which these viral proteins damage the vascular endothelium in HIV patients. A better understanding of the molecular mechanisms of HIV protein associated endothelial dysfunction leading to cardiovascular disease is likely to be pivotal in devising new strategies to treat and prevent cardiovascular disease in HIV-infected patients.

## Introduction

The introduction of highly active antiretroviral therapy has lead to a drastic reduction in viral burden and opportunistic infections, resulting in a remarkable improvement in the life expectancy of HIV-infected individuals. However, it is now evident that HIV-infected individuals have an enhanced susceptibility to non-AIDS (Acquired Immunodeficiency syndrome)-related comorbidities such as cardio-vascular diseases (CVDs), which have emerged as prominent causes of morbidity and mortality in this population (1–9). Atherosclerotic CVD rates and risk of myocardial infarction are significantly elevated in HIV-infected individuals than the general population (1, 10, 11). Studies further indicate that both clinical cardiovascular events such as coronary heart disease (11–13), peripheral artery disease (14), as well as subclinical cardiovascular damage such as elevation of intima-media thickness (15, 16), coronary calcification (17), abnormal ankle-brachial index (18) and silent myocardial ischemia (19) are much higher in HIV-infected individuals (20).

## Etiopathogenesis of Cardiovascular Disease in Patients With HIV Infection

The increased cardiovascular risk in HIV-infected individuals is attributable to a combination of multiple factors, including higher prevalence of traditional risk factors, inherent effects of the HIV infection, effects of antiretroviral therapy and the presence of other co-morbidities seen frequently in HIV-positive patients (such as hepatitis C virus and herpes family virus co-infections). Though initial studies indicated a predominance of traditional CVD risk factors (21, 22) and effect of ART (23) as major causes for CVD among HIV-positive individuals (10, 24, 25), evidence from experimental and observational studies (26, 27) in recent years have redirected attention more toward the consequences of HIV infection itself. Hsue et al demonstrated a correlation between HIV infection and premature atherosclerosis even in the absence of detectable viremia, immunodeficiency, and ART exposure, with the atherosclerosis being independent of traditional cardiovascular risk factors (28).

Among multiple pathogenic effects that contribute to atherosclerosis and ultimately CVD, HIV-induced endothelial dysfunction is now established as a major contributing factor. Higher plasma HIV RNA levels have been shown to correlate with endothelial dysfunction in HIV-infected patients (29). A transgenic mouse model expressing HIV viral proteins env, tat, nef, vpu, vpr, and rev demonstrated aortic endothelial dysfunction and increased arterial stiffness (30). HIV-infected patients had significantly impaired endothelial function, as demonstrated by reduced flow-mediated dilation, a measure of endothelial vasomotor function in comparison to the HIV-negative group (31).

## Endothelial Dysfunction and Cardiovascular Disease

Endothelial dysfunction as a precursor of atherosclerosis and future cardiovascular events has been demonstrated in multiple population studies (32, 33). The development of atherosclerosis resulting from dysfunctional endothelium is highly complex and regulated by several factors. Endothelial dysfunction is characterized by decreased anti-oxidant, anti-inflammatory and anti-thrombotic properties (due to reduced NO bioavailability) and increased endothelial permeability, pro-inflammatory cytokine levels, and adhesion molecule expression. Leukocyte recruitment and adhesion represent the initial events in development of atherosclerosis. Leukocyte recruitment is mediated by several chemoattractants such as IL-6, IL-8, and MCP-1 and adherence of leukocytes to the endothelium is mediated by cell adhesion molecules (CAM). Leukocytes, especially monocytes traverse the endothelium, and migrate into the intima (34). Transmigration of leukocytes, as well as infiltration of plasma contents into the vascular wall is facilitated by an increase in endothelial permeability. These infiltrated plasma contents such as modified low-density lipoprotein (LDL), along with substances produced by infiltrated leukocytes, such as cytokines and chemokines, alter smooth muscle function and contribute to the development of atherosclerosis (35). Further, in the intima, the monocytes differentiate into macrophages, expressing receptors that facilitate lipid uptake. On lipid uptake and accumulation, macrophages transform into foam cells. These foam cells initiate atherosclerotic lesions, which are later characterized by plaque formation (34). Studies suggest that HIV impairs several of these processes that maintain vascular homeostasis, potentially leading to atherosclerosis (Table 1). Several mechanisms have been suggested to explain how HIV infection induces endothelial dysfunction leading to CVD, including direct HIV infection of endothelial cells (ECs), inflammation and effect of HIV proteins HIV proteins released in the endothelial microenvironment or directly transferred to ECs by HIV and HIV-infected cells represent critical mediators of endothelial dysfunction. This article reviews the current understanding of the mechanisms by which HIV, in particular, the different HIV proteins drive EC dysregulation, potentially leading to CVD.

**Table 1:** Summary of the potential mechanisms by which HIV protein-induced endothelial dysfunction contribute directly or indirectly to the development of atherosclerosis and CVD.

**HIV protein**	**Endothelial dysfunction^**a**^**	**Association with cardiovascular disease**
Gp120 Tat Nef	↑Apoptosis (36–43)	Promotes atherosclerotic plaque formation and plaque instability
Gp120 Tat Nef	↑IL-6 (44–46)	Increases intima media thickness Monocyte/macrophage recruitment Stimulates synthesis of acute phase proteins (CRP)
Gp120	↑IL-8 (47)	Leukocyte recruitment Mediates release of MCP-1
Tat	↑IL-1β (48)	Induces macrophage/foam cell apoptosis, Increased expression of pro-inflammatory cytokines Increased expression of adhesion molecules Migration of vascular smooth muscle cells and ECs
Tat Nef	↑MCP-1 (49, 50)	Increases monocyte recruitment
Gp120	↑ET-1 (51)	Increased smooth muscle proliferation and migration
Gp120 Tat Nef	↑ICAM-1 (52–54)	Adherence and transmigration of leukocytes into the vessel wall
Tat	↑VCAM-1 (48, 55)	Adherence and transmigration of leukocytes into the vessel wall
Tat	↑E-selectin (48)	Initial rolling of leukocytes on ECs
Gp120 Tat	↑ Endothelial permeability (47, 51, 56–59)	Facilitates infiltration of leukocytes and plasma contents into vessel wall
Gp120	↑ MMP-2, ↑ MMP-9 (60)	Facilitates endothelial damage leading to unstable plaque formation
Gp120	↓ NO levels (61, 62)	Abnormal vascular tone regulation and enhanced platelet adhesion and aggregation
Gp120 Tat	↑ROS (55, 56, 63, 64)	Increased foam cell formation leading to plaque growth

a *References are in parenthesis*.

## HIV Encoded Proteins and Endothelial Dysfunction

HIV is a retrovirus with a glycoprotein-rich envelope surrounding a nucleocapsid. The HIV structural and regulatory/accessory proteins are designed for the virus to adapt efficiently to the human host, thereby promoting its replication and transmission. The HIV viral genome contains 9 principal genes, gag, pol, env, tat, rev, vpu, vpr, vif, and nef. The Gag-Pol precursor protein undergoes proteolytic cleavage to generate the matrix p17, capsid p24, nucleocapsids p9 and p6, reverse transcriptase, protease, and integrase, all of which are major structural components of the viral core. The Env undergoes proteolytic cleavage to generate the envelope glycoproteins gp120 and gp41. Tat and Rev are the regulatory proteins, while Vpu, Vpr, Vif, and Nef are the accessory proteins (65). Among these viral proteins, gp120, Tat and Nef play a major role in the pathogenesis of endothelial dysfunction. The experimental evidence supporting a functional role for the HIV viral proteins in the disruption of EC cell biology is outlined in the following sections.

### HIV gp120

HIV envelope glycoprotein is synthesized as a precursor glycoprotein, gp160, which is then processed into an amino terminus subunit, gp120, and a carboxyl transmembrane subunit, gp41. The envelope glycoprotein, gp120 is expressed on the outer layer of the virus, as well as on the surface of infected cells. Gp120 is critical for virus infection, as the protein is necessary for binding to specific cell surface receptors on target cells and facilitating virus entry. The primary receptor for gp120 is the CD4 receptor, while the main co-receptors are CXCR4 and CCR5. Gp120 is found both in the free form in the body fluids of HIV-positive patients (66, 67) and bound form on the surface of apoptotic CD4 positive T-cells (68). In fact, gp120 has been shown in the germinal center of lymph nodes in HIV-infected individuals under ART with no detectable viral replication (69). Multiple studies have confirmed that gp120, both in soluble and surface bound form, has an important role in viral pathogenesis on diverse uninfected bystander cells (70–74), including ECs.

Gp120 is associated with apoptosis, adhesion molecule expression, pro-inflammatory cytokine production and EC permeability. Gp120 present either on viral particles, surface of infected cells, or as free soluble protein causes endothelial apoptosis predominantly by direct interaction with the co-receptor, CXCR4. Gp120 induces apoptosis in human coronary ECs (36), human umbilical vein ECs (HUVECs) (37, 38), lung microvascular ECs (LMVECs) (39), and brain microvascular ECs (BMVECs) (40, 41). EC apoptosis is an important process, initially in atherosclerotic plaque formation, and later in the progression to an advanced stage of atherosclerosis, when the plaques become vulnerable to rupture (42, 75, 76). The molecular mechanism by which gp120 exerts its endothelial toxicity, may involve caspase-3 activation (38), Bax upregulation (38), protein kinase C (PKC) activation (77) and p38 mitogen-activated protein (MAP) kinase signaling (41). Gp120 also induces an increase in reactive oxygen species (ROS), signaling oxidative stress to the ECs (56, 63). Oxidative stress induced by generation of excess reactive oxygen species is a critical process in the development of atherosclerosis (78). HIV-induced ROS likely contributes to endothelial dysfunction through direct effects on the endothelium and/or indirectly through monocytes /macrophages contacting the vessel wall. The viral glycoptotein is also able to increase endothelin-1 (ET-1) secretion (51) and promote surface expression of Endothelial monocyte activating polypeptide II (EMAPII) (79). ET-1 mediates the reduction of vascular nitric oxide production by ECs, leading to the smooth muscle proliferation and migration, which in turn leads to arterial vasoconstriction (80), whereas EMAPII is released in response to stress such as hypoxia, mechanical strain and apoptosis (81) and acts as a pro-apoptotic factor. In addition, a recent study has shown that HIV gp120 (X4 and R5) promotes EC senescence and impairs the regulation of senescence-associated microRNAs (82). Senescent ECs develop a dysfunctional phenotype acquiring pro-inflammatory, pro-oxidant, vasoconstrictor, and prothrombotic properties (83).

Gp120 is directly involved in upregulation of pro-inflammatory cytokines such as IL-6 and IL-8 in primary ECs (44). IL-6 and IL-8 play a major role in recruitment of leukocytes, especially of the monocyte/macrophage and neutrophil lineage, respectively. IL-6 can also actively promote atherogenesis, both directly by inducing vascular endothelial dysfunction, extracellular matrix degradation and indirectly by stimulating hepatocytes synthesis of acute phase proteins involved in inflammation, such as C-reactive protein (84). Gp120 also facilitates monocyte (52) and T-cell adherence (85) to the vascular endothelium through upregulation of CAMs. Among the CAMs, E-selectin is involved in the initial rolling of leukocytes on the endothelial cells, while ICAM-1 and VCAM-1 induce firm adhesion and transmigration of leukocytes across the endothelium (86). Gp120 augments expression of ICAM-1, but not VCAM-1 or E-selectin, in ECs of multiple origins, including human coronary artery, lung, brain, umbilical vein, and dermal microvascular ECs (52).

Gp120 also increases endothelial permeability by various mechanisms including cytoskeletal rearrangement (56), down-regulation of tight junction proteins (51) and PKC activation (47). An increase in endothelial permeability was observed in brain endothelial cultures of HIV gp120 transgenic mice (87), compared to non-transgenic mice. Gp120 also induces expression of the matrix metalloproteases (MMPs), MMP-2 and MMP-9, that mediate endothelial damage with the formation of an unstable atherosclerotic plaque morphology (60). Additionally, gp120 reduces the EC-derived nitric oxide (NO) synthesized by the NO synthase, thus affecting endothelium-dependent vasorelaxation and enhancing platelet adhesion and aggregation (61, 62).

### HIV Tat

HIV Tat (trans-activator of transcription) is a regulatory protein encoded by the tat gene that enhances viral transcription (88). Tat has been detected in the sera of HIV patients (89), even during complete ART (cART) (90). Tat is secreted into the extracellular microenvironment by HIV-infected T-cells and monocyte/macrophages (89, 91). In the circulation, Tat is suggested to act as a proto-cytokine, modulating the functions of several cells including ECs (92). Thus, Tat is involved in the pathogenesis of several HIV-associated disease conditions ranging from pulmonary hypertension to cognitive abnormalities (36, 48, 93–95). Tat protein possesses both transcription promotion and membrane transduction properties. Tat has five discrete domains, the N-terminal, cysteine-rich, core, basic, and C-terminal domain. Tat interacts with three known receptors to trigger endothelial dysfunction. The C-terminal domain, containing an Arg-Gly-Asp (RGD) sequence, binds with high affinity to the integrins alphaVbeta1 and alphaVbeta3 receptors (96). The basic domain binds to the integrin alphaVbeta5 receptor (97) as well as the Flk-1/KDR receptor (98). Tat activates these receptors to initiate endothelial signaling pathways that affect diverse processes such as endothelial permeability (57, 58), cytokine production (59), adhesion (48), angiogenesis (99–102), and apoptosis (43).

Tat exhibits a dual function with regard to survival regulation, exhibiting either EC proliferation or apoptosis, depending on the micro-environment conditions (103). One of the prominent properties of Tat is that of a direct angiogenic factor (92). Endothelial proliferation is enhanced by factors such as FGF-2 (fibroblast growth factor) (104). Tat activates Rac1 through a signaling cascade involving RhoA, Ras, and extracellular signal-regulated kinase (ERK), which in turn, induces EC proliferation and survival (105). Tat mediates Rac1 activation through PAK-1, phosphorylates c-Jun N-terminal kinase (JNK), activates endothelial NADPH oxidase and regulates actin cytoskeletal dynamics (106). Tat has been suggested to play a role in HIV-related Kaposi sarcoma by promoting EC proliferation and tumor angiogenesis, where Tat binds specifically and activates the Flk-1/kinase insert domain receptor (Flk- 1/KDR), a VEGF-A tyrosine kinase receptor, and promotes angiogenesis (98). Contrary to its role in angiogenesis, Tat also induces the apoptosis of primary microvascular ECs via either TNF-alpha secretion or through activation of the Fas-dependent pathway (43). Fiala et al. (36) analyzed the pathogenesis of HIV-related cardiomyopathy, and found that exogenous Tat protein was capable of activating apoptosis of both ECs or cardiomyocytes. A recent report indicates that HIV Tat along with morphine induces autophagy in pulmonary ECs, suggesting a role for Tat in HIV-related pulmonary arterial hypertension in the presence of opioids (107). In addition, Tat also promotes EC senescence and dysregulation of senescence-associated microRNAs (82).

Tat stimulates the release of pro-inflammatory cytokines and induces expression of CAMs (45, 48, 55, 108, 109) in ECs of diverse origin (i.e., pulmonary artery, umbilical vein, aorta, and brain). In human vascular ECs (HUVECs), Tat stimulates the upregulation of inflammatory mediators, including IL-1β, MCP-1, VCAM-1 and E-selectin through nuclear factor-kappa B (NF-κB) (48, 108). IL-1β can induce macrophage and foam cell apoptosis, causing the release of their lipid content into the intima of the artery and contributing toward the lipid core in the plaque (110). IL-1β can also induce the expression of cytokines, adhesion molecules and the migration and mitogenesis of vascular smooth muscle and endothelial cells (111). MCP-1 is a major chemokine involved in monocyte recruitment during atherosclerosis development (35). Expression of IL-6 and MCP-1 is dependent upon the activity of the kinases, PKC (45) and cAMP-dependent protein kinase A (49). Tat stimulated ICAM-1 expression in HUVECs by suppressing miR-221/-222 via an NF-κB-dependent pathway (53), while Tat stimulated VCAM-1 expression through p38 MAP kinase and NF-kB activation (55). Upregulation of these adhesion molecules resulted in monocyte (45, 108, 112) and T-cell (113) adhesion to the endothelium. Furthermore, Matzen et al showed that Tat in combination with TNF-alpha, a cytokine increased in sera and tissues of HIV-infected patients, acts synergistically to increase the adhesion of leukocytes to ECs, suggesting that both these proteins act in co-operation to contribute to the vascular damage during HIV infection (113). Finally, Tat induces endothelial oxidative stress through activation of NADPH oxidase and through decreased antioxidant capacity. Tat-induced MAPK signaling requires upstream superoxide production by various NADPH oxidase subunits. Moreover, Tat-induced ROS activates the NF-kB pathway (55) and decreases GSH levels (64). Tat also attenuates the expression of the mitochondrial superoxide scavenger, Manganese-superoxide dismutase (Mn-SOD) (46, 114).

### HIV Nef

HIV Nef is a 27-kD, n-myristoylated accessory protein that lacks enzymatic activity. It is an adaptor molecule containing multiple domains essential for interaction with host cell signaling molecules (115, 116). Nef is involved in modulation of several intracellular functions that include regulation of protein trafficking and cell signaling pathways, attenuation of antibody maturation in B cells (117), and increase in HIV infectivity (118). The presence of Nef has been shown in the endothelium of coronary and pulmonary arteries of SIV-HIV-Nef-infected macaques (50). Sowinski et al (119) demonstrated that Nef induces the formation of conduit-like nanotubes, connecting HIV-positive cells to bystander cells. Further, Wang et al. (50) showed that Nef transfer from HIV-infected cells to ECs promotes endothelial dysfunction (50, 120). Nef is also delivered to bystander cells through exosomes (121). ECs, especially those present in developing atherosclerotic plaques, would therefore be in a prime physical position to receive Nef transfer from circulating monocytes and T cells. Transgenic mice that express CD4-promoter-driven Nef develop multiple pathologies including vasospasm in the heart (122). Studies show that Rhesus macaques demonstrate pulmonary hypertension (PH)-like pulmonary vascular remodeling, when infected with chimeric SHIVnef virions, but not with SIV, indicating a role of HIV-Nef in PH, with certain Nef gene variants showing a higher propensity to develop PH (123, 124).

Similar to gp120 and Tat, Nef has been associated with several aspects of HIV-induced endothelial dysfunction. Acheampong et al. (42) showed that Nef, when expressed both extracellularly and endogenously, induces apoptosis in primary human brain microvascular ECs (HB-MVECs) by activation of caspases. A microarray analysis of apoptosis genes in Nef-transduced HB-MVECs demonstrated that the up-regulated genes belong to both mitochondrial and Fas/FasL apoptotic pathways, indicating that Nef may utilize multiple pathways to induce apoptosis in ECs. In contrast, in the context of Kaposi's sarcoma, Nef and KSHV oncogene K1 synergistically promote angiogenesis by inducing cellular miR-718 to regulate the PTEN/AKT/mTOR signaling pathway. However, in Kaposi's sarcoma, Nef in combination with KSHV oncogene K1 synergistically induces cellular miR-718 to regulate the PTEN/AKT/mTOR signaling pathway and thus promotes angiogenesis. This pathway is an important factor in aberrant neovascularization caused by KS-associated herpesvirus (KSHV) (125).

Nef-expressing T cells demonstrate enhanced adherence to ECs as observed by their impaired diapedesis and migration into the subendothelial space (126). Fan et al have shown ERK kinase-mediated ICAM-1 upregulation in vascular ECs stably expressing Nef (54). Furthermore, Nef increases endothelial MCP-1 production through activation of the NF-kB signaling pathway (50). In a recent study, Nef was shown to be involved in the alteration of EC cholesterol homeostasis through phosphorylation of Caveolin-1 (Cav-1), leading to Cav-1 redistribution and impairment of HDL-mediated cholesterol efflux in ECs (127). In addition to its direct effects on ECs, Nef activates macrophages and produces foam cells (128). The interactions of these foam cells with ECs could also contribute to EC dysfunction, and potentially facilitate the development of atherosclerosis.

## Conclusion and Future Perspectives

**Figure 1:**
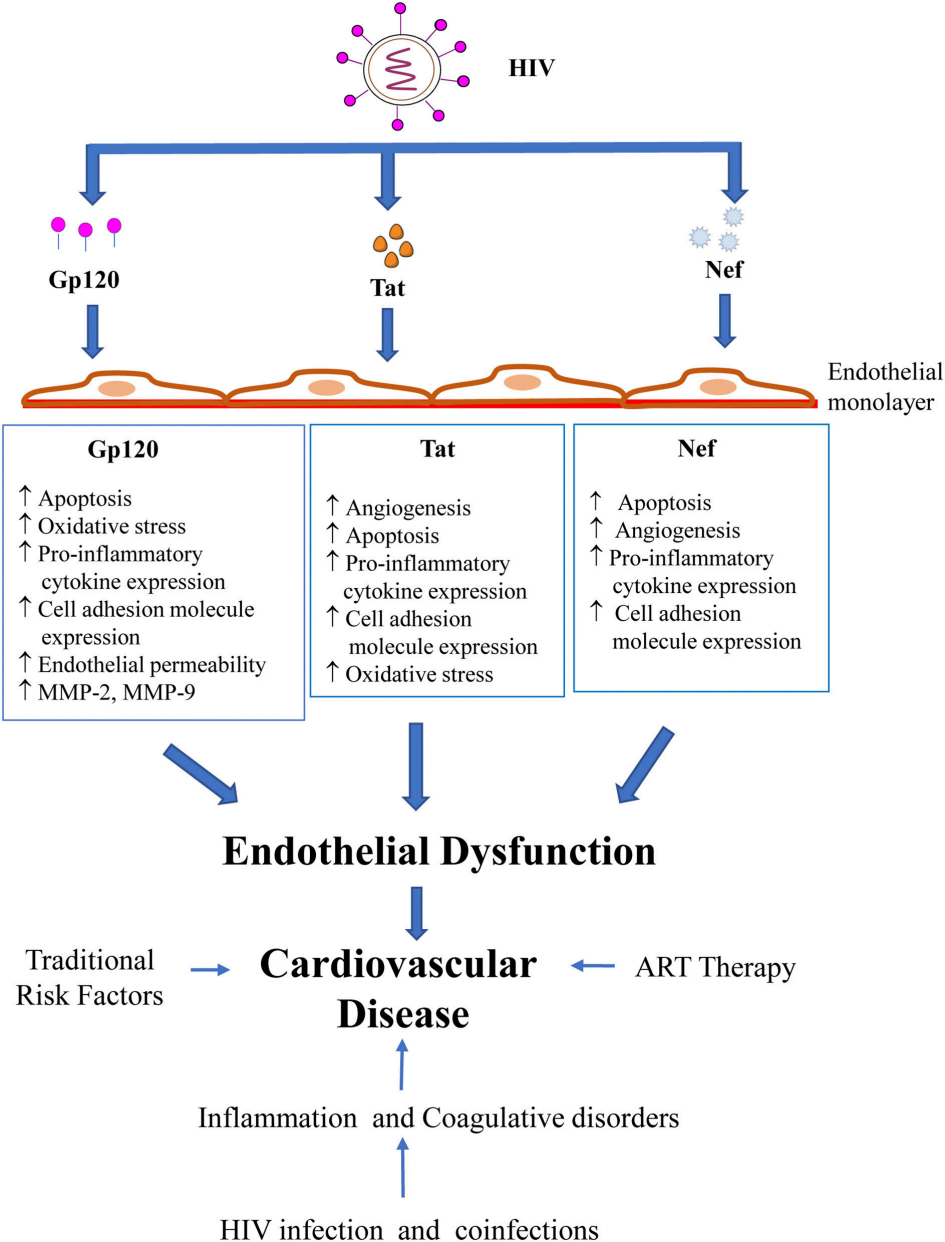
HIV proteins and their effects on endothelial dysfunction.

In summary, the present review underscores the role of HIV-encoded proteins, specifically Gp120, Tat and Nef, in the pathogenesis of endothelial dysfunction, a precursor for the development of CVD (Figure 1). Our understanding of the complex interaction of traditional factors, inflammation and immune activation, cART and HIV in the progression of CVD has grown rapidly over the past decade. However, a more detailed exploration into the mechanisms of HIV-induced endothelial dysfunction is needed to formulate targeted approaches to prevent and treat HIV-related vascular diseases. Presently, large prospective studies such as REPRIEVE (NCT02344290), a randomized trial to prevent vascular events in HIV, are being carried out that will provide valuable data on the relation between inflammation, CVD and HIV infection (129). Research efforts will also need to focus on identifying HIV-specific markers that could predict the risk of developing CVD and facilitate the early detection of CVD in HIV patients. An accurate assessment of patients based on such biomarkers could be incorporated in guidelines such as the European AIDS Clinical Society guidelines (130) on the joint management and prevention of CVD in HIV patients, thereby providing vital information to guide clinicians on the most appropriate approach to prevent and treat CVD in this high-risk population.

## Author Contributions

GR and DP wrote the manuscript. AA wrote and revised the manuscript.

### Conflict of Interest Statement

The authors declare that the research was conducted in the absence of any commercial or financial relationships that could be construed as a potential conflict of interest.
